# Comparison of Emotional Dysregulation Features in Cyclothymia and Adult ADHD

**DOI:** 10.3390/medicina57050489

**Published:** 2021-05-12

**Authors:** Giulio Emilio Brancati, Margherita Barbuti, Elisa Schiavi, Paola Colombini, Martina Moriconi, Alessandro Pallucchini, Marco Maiello, Giulia Menculini, Giulio Perugi

**Affiliations:** 1 Psychiatry 2 Unit, Department of Clinical and Experimental Medicine, University of Pisa, Via Roma 67, 56126 Pisa, Italy; giuliobrancati@gmail.com (G.E.B.); margherita.barbuti@hotmail.com (M.B.); p.colombini@hotmail.it (P.C.); martinamoriconi@hotmail.it (M.M.); pallucchini.a@gmail.com (A.P.); marcomaiello@aol.com (M.M.); 2 Psychiatry 2 Unit, Azienda Ospedaliero-Universitaria Pisana, Via Roma 67, 56126 Pisa, Italy; el.schiavi@gmail.com; 3 Division of Psychiatry, Department of Medicine, University of Perugia, Piazzale Lucio Severi 1, 06132 Perugia, Italy; giuliamenculini@gmail.com

**Keywords:** attention-deficit/hyperactivity disorder, cyclothymia, emotional dysregulation

## Abstract

*Background and Objectives*: Emotional dysregulation is central to the problem of the overlap between attention-deficit/hyperactivity disorder (ADHD) and cyclothymia. The aim of the study was to evaluate comorbidity rates between ADHD and cyclothymic disorder and to explore demographic and clinical differences among the groups, focusing on affective temperament and emotional dysregulation. *Materials and Methods*: One hundred sixty-five outpatients attending the Second Psychiatry Unit at the Santa Chiara University Hospital (Pisa) were consecutively recruited: 80 were diagnosed with ADHD, 60 with cyclothymic disorder, and 25 with both conditions. Temperament Evaluation of Memphis, Pisa, Paris, and San Diego (TEMPS-M) and the 40-item version of Reactivity, Intensity, Polarity, and Stability questionnaire (RI-PoSt-40) were administered. *Results*: Cyclothymic patients were more frequently female and older with respect to the ADHD groups. Both comorbid and non-comorbid ADHD patients showed significantly lower educational attainment and more frequently had substance use disorders. Panic disorder was common in non-comorbid cyclothymic patients, who showed significantly higher rates of familial panic disorder, major depressive disorder and suicide attempts in comparison with patients only diagnosed with ADHD. Cyclothymic patients without ADHD were also characterized by fewer hyperthymic temperamental traits, higher depressive and anxious dispositions, and a greater negative emotionality. No significant differences among groups were observed for cyclothymic temperament and overall negative emotional dysregulation, but comorbid patients with both conditions scored the highest in these subscales. This group also showed significantly higher affective instability with respect to ADHD patients without cyclothymia and was less frequently diagnosed with bipolar disorder type II than patients from both the other groups. *Conclusions*: ADHD and cyclothymia often co-occur and show similar levels of emotional dysregulation. However, cyclothymic patients may be more prone to negative emotionality in clinical settings. Subjects with “sunny” cyclothymic features might escape the attention of clinicians unless ADHD is present.

## 1. Introduction

The failure to regulate emotions, namely emotional dysregulation, is a novel dimension of interest in psychiatric research, which characterizes a multitude of conditions usually observed in clinical practice. Emotional dysregulation shows heterogeneity in its nomenclature, definition, and presentation and can be broadly defined as “rapid oscillations of intense affect, with a difficulty in regulating these oscillations or their behavioural consequences” [1]. Issues in differential diagnoses between attention-deficit/hyperactivity disorder (ADHD) and bipolar spectrum disorders often involve emotional dysregulation [2,3].

ADHD is a neurodevelopmental disorder characterized by symptoms of inattention, hyperactivity, and impulsivity, which first appears during childhood and, in approximately half of the cases [4], persists into adulthood. Since problems with regulation of emotions are longitudinally associated with increased psychiatric comorbidity and ADHD persistence [5,6], up to 70% of adults diagnosed with ADHD also show emotional dysregulation [7]. As a consequence, different authors consider it as a core feature of adult ADHD [8,9,10].

At the same time, emotional dysregulation has been long considered a distinctive constitutional trait underlying cyclothymic temperamental variants associated with bipolar affective disorders [11]. Cyclothymic temperament, indeed, has been operationally defined as a constellation of habitual subsyndromal biphasic mood swings with typical abrupt shifts from one phase to another, associated with both subjective and behavioral manifestations, pertaining to interepisodic and premorbid periods of affective disorders [12]. Cyclothymic temperament has been shown to negatively affect illness course [13,14,15] and in its extreme presentations actually constitutes a disabling mood disorder on its own [16]. In these patients, mood reactivity and emotional dysregulation represent the core symptomatology, often beginning very early in childhood [11].

Both ADHD and bipolar spectrum disorders share a difficulty in modulating emotionally driven behavioral responses. Accordingly, a plausible common neurophysiological basis has been hypothesized, involving neurodevelopmental dysfunctions of amygdala and fronto-limbic circuitries [7,17,18]. However, studies assessing the comorbidity between ADHD and cyclothymic disorder are lacking, and it has not been investigated whether ADHD and cyclothymia share the same manifestations of emotional dysregulation or single facets could distinguish among these disorders.

The recently developed Reactivity, Intensity, Polarity, and Stability questionnaire could represent a useful self-report tool to quantify emotional dysregulation in some of its different facets both in psychiatric patients and healthy subjects [19]. We recently validated a 40-item version of the scale (RIPoSt-40), which includes four subscales measuring affect oscillation over time (affective instability), tendency for more intense and frequent negative and positive feelings (negative and positive emotionality), and inability to regulate impulsive behavioral responses to emotionally salient stimuli (emotional impulsivity) [20]. Both patients affected by ADHD and patients affected by cyclothymic disorder were included among validation samples, and marginally significant differences between the groups emerged in emotional impulsivity, with higher scores in those with ADHD, while both clinical groups shared large deviations from the non-clinical sample [20]. However, comorbidity between ADHD and cyclothymia was not taken into consideration.

The first aim of the present study was to evaluate comorbidity rates between ADHD and cyclothymic disorder in outpatients attending the Psychiatry 2 Unit at Santa Chiara University Hospital in Pisa. Second, we explored demographic and clinical differences among patients diagnosed with ADHD, cyclothymia, or both disorders. Special attention was given to affective temperamental dispositions and emotional dysregulation facets.

## 2. Materials and Methods

According to a naturalistic approach, patients were consecutively enrolled between January 2018 and June 2019 at the Outpatient Service of the Psychiatry 2 Unit of Santa Chiara University Hospital (Pisa, Italy). All subjects provided written informed consent for the study participation. The study was carried out in accordance with The Code of Ethics of the World Medical Association (Declaration of Helsinki), and the study protocol was approved by the Ethical Committee of the University of Pisa on 15 March 2018 (N. 12712_PERUGI).

Adult patients (age ≥ 18 years) receiving a diagnosis of ADHD in accordance with structured Diagnostic Interview for Adult ADHD, second edition (DIVA 2.0), and/or meeting criteria A-B and D-G for cyclothymic disorder from the Diagnostic and Statistical Manual of Mental Disorders, Fifth Edition (DSM-5), were included in the study. Exclusion criterion C for cyclothymic disorder was not applied, and a lifetime history of major mood episodes, whether depressive or (hypo)manic, did not rule out the diagnosis. In contrast, patients presenting with current major depressive or (hypo)manic episodes were excluded. This approach permitted the inclusion of patients with a cyclothymic background, otherwise diagnosed with major depressive, bipolar II, or bipolar I disorder, who experience frequent mood shifts, mild to moderate symptoms, and significant distress and functional impairment. In addition, an early undetermined onset of cyclothymia (<21 years old) was considered mandatory, in accordance with Akiskal and Mallya criteria for cyclothymia [12], to exclude patients with iatrogenic or residual mood swings. Patients with intellectual disability and/or schizophrenia spectrum disorders according to DSM-5 criteria were excluded from the samples. 

Socio-demographic variables and lifetime psychiatric comorbidity according to DSM-5 criteria were assessed in a single consultation by the participating psychiatrists. Affective temperamental traits were measured by means of the Temperament Evaluation of Memphis, Pisa, Paris, and San Diego (TEMPS-M) [21,22], a self-evaluation form of 35 items coded on a 5-point Likert scale (from absent to very much) and including five subscales, one for each affective temperamental disposition, namely depressive, cyclothymic, hyperthymic, irritable, and anxious. Emotional dysregulation was measured using the Reactivity, Intensity, Polarity, and Stability questionnaire in its 40-item version (RIPoSt-40) [20]. Items rated on a Likert scale ranging from 1 (“never”) to 6 (“always”) were summed to compute four subscales scores, assessing affective instability, positive and negative emotionality, and emotional impulsivity, and a second-order negative emotional dysregulation score made up of affective instability, negative emotionality, and emotional impulsivity subscales. A new index was also computed as the ratio between negative and positive emotionality subscale scores (NE/PE ratio).

All the statistical analyses were performed using IBM SPSS Statistics for Mac, Version 25.0 (SPSS Inc., Chicago, IL, USA). Demographic and clinical variables as well as RIPoSt-40 and TEMPS-M scores were compared among the groups. Descriptive statistics (mean, standard deviation (SD)) were used to describe characteristics of the subsamples. Comparative analyses were conducted using chi-square test for categorical variables (with z-test contrasts and Bonferroni’s correction) and one-way analysis of variance (ANOVA) for continuous variables (with a posteriori contrasts according to Scheffé’s procedure). Finally, a principal component analysis (PCA) with varimax rotation was performed on TEMPS-M and RIPoSt-40 subscales. All the components with an eigenvalue greater than 1 were retained, according to Kaiser criterion [23], and compared among the groups. The significance level in all statistical tests was set to 0.05.

## 3. Results

A sample of 165 patients was recruited: 80 of them were diagnosed with ADHD, 60 with cyclothymic disorder, and 25 with both ADHD and cyclothymic disorder. Significant differences in age, gender, and educational level were observed among these groups (Table 1). Cyclothymic patients without ADHD showed a significantly higher age in comparison with patients only diagnosed with ADHD, while comorbid patients had intermediate age and did not significantly differ from both the other groups. Instead, all the groups significantly differed from each other in terms of gender proportions. Women were overrepresented in the cyclothymic group without ADHD and underrepresented in the ADHD group, with an intermediate rate in comorbid ADHD–cyclothymic patients. Both comorbid and non-comorbid ADHD patients showed significantly lower educational attainment as compared with non-comorbid cyclothymia: university or superior educational level was more frequently observed in cyclothymic patients than in the other groups, while both ADHD and ADHD–cyclothymic patients more frequently showed a primary or middle school educational level as compared with cyclothymic patients.

Bipolar disorders were common among all the groups, being diagnosed in 83 of 165 patients (50.3%). No significant differences in bipolar disorder type I prevalence were found among the groups. However, bipolar disorder type II was more frequently diagnosed in non-comorbid ADHD and cyclothymia than in comorbid ADHD–cyclothymic patients. Substance use disorders were significantly more frequently observed in both the ADHD groups, which showed a prevalence approximately twice that reported in non-comorbid cyclothymic patients (63.8% vs. 28.3%). The opposite pattern was observed for panic disorder, though not significantly (*p* = 0.060): in patients with cyclothymia, whether comorbid with ADHD or not, panic disorder was almost twice as prevalent as in patients only affected by ADHD (28.2% vs. 15.0%).

Approximately a half of patients had a first-degree family history of bipolar disorder (75 of 165, 45.5%), without significant differences among the groups. As for the other disorders, first-degree family history showed patterns that were similar to those observed for psychiatric comorbidity. On one hand, the highest rate of familial substance use disorders was found in ADHD patients, and the lowest was found in non-comorbid cyclothymic patients. Comorbid ADHD–cyclothymic patients showed an intermediate rate. However, these differences failed to reach statistical significance (*p* = 0.064). On the other hand, familial panic disorder was significantly more frequent in non-comorbid cyclothymic patients than in non-comorbid ADHD, while comorbid patients had an intermediate rate and did not significantly differ from both the other groups. Importantly, a similar pattern of significant differences was found for familial major depressive disorder and suicide attempts.

While no significant differences were found for cyclothymic and irritable temperamental dimensions, depressive, hyperthymic, and anxious temperament scores from TEMPS-M significantly differed among groups (Table 2). Patients with cyclothymia without ADHD obtained significantly higher scores than patients only diagnosed with ADHD on both the depressive and anxious temperament subscales. Conversely, the hyperthymic temperament score was significantly higher in non-comorbid ADHD as compared with non-comorbid cyclothymia. The comorbid ADHD-cyclothymia group showed intermediate scores of depressive, anxious, and hyperthymic temperaments but the highest scores of cyclothymic and irritable temperaments among the groups.

**Figure 1:**
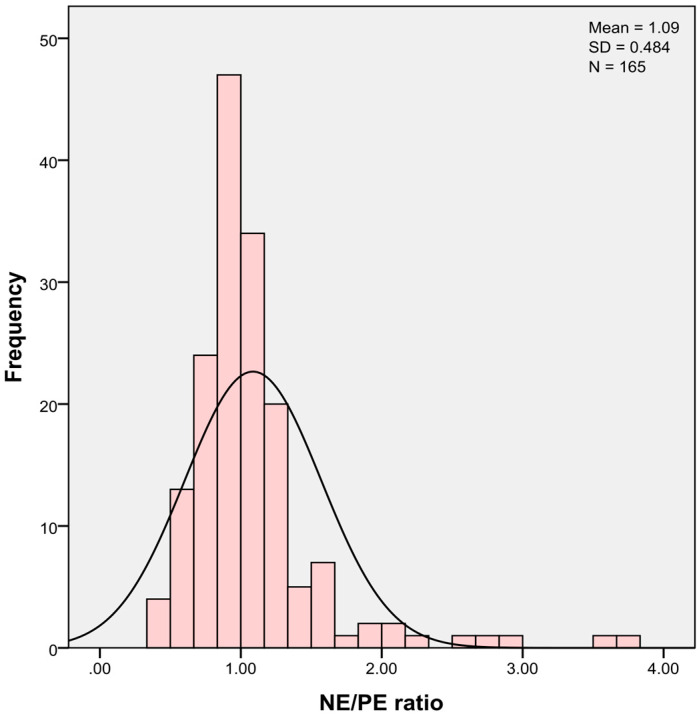
Distribution of NE/PE ratio in the sample. Abbreviations: NE/PE = negative emotionality/positive emotionality; SD = standard deviation.

Among emotional dysregulation facets measured by RIPoSt-40, affective instability and negative emotionality significantly differed among the groups, while positive emotionality, emotional impulsivity, and overall negative emotional dysregulation did not show significantly different scores. As for affective instability, comorbid patients with ADHD and cyclothymia scored significantly higher than patients with only ADHD. Negative emotionality, instead, was significantly higher in cyclothymic patients without ADHD as compared with non-comorbid ADHD patients. As for NE/PE ratio, a significant deviation from balanced emotional polarities emerged in the whole sample, with a prominence of negative over positive emotionality using one-sample *t*-test (test = 1, t = 2.29, *p* = 0.023; Figure 1). As for negative emotionality, NE/PE ratio was significantly higher in cyclothymic patients without ADHD as compared with non-comorbid ADHD patients.

Finally, two components, explaining 63.74% of the variance, were extracted through PCA with varimax rotation performed on TEMPS-M and RIPoSt-40 subscales (Table 3). Cyclothymic, depressive, and anxious temperaments were mainly loaded together with affective instability, negative emotionality, and emotional impulsivity in the first component, which explained 45.18% of the variance and was interpreted as representing negative affect dysregulation. Hyperthymic temperament and positive emotionality were mainly loaded in the second component, which explained 18.56% of the variance and was interpreted as representing positive affect. Irritable temperament was mainly loaded in the first component but also showed a medium loading in the second one. Significant differences among diagnostic groups were found in both the negative (F = 4.36, *p* = 0.014) and positive components (F = 8.71, *p* < 0.001). Patients only diagnosed with cyclothymia scored significantly higher on the first component than patients with only ADHD (z-scores (mean ± SD): 0.23 ± 0.98 vs. −0.23 ± 0.96), while comorbid patients with both diagnoses did not significantly differ from the others (z-score (mean ± SD): 0.18 ± 1.04). Conversely, both comorbid and non-comorbid ADHD groups had significantly higher scores for positive affect than patients with non-comorbid cyclothymia. The highest score was found in patients with both ADHD and cyclothymia, followed, in order, by ADHD and cyclothymia (z-scores (mean ± SD): 0.34 ± 1.03; 0.20 ± 0.91; −0.40 ± 0.97).

## 4. Discussion

In our study, we consecutively recruited adult psychiatric outpatients diagnosed with ADHD and/or cyclothymic disorder. Importantly, a high diagnostic overlap among categories was observed. Indeed, approximately one-fourth of all the ADHD patients included in the study also presented with cyclothymic disorder (25 of 85, 23.8%). This latter rate doubles the lifetime prevalence of bipolar disorder in ADHD samples, which approximately settles to 10% according to recent estimates [24]. A selection bias could be assumed, given that the sample was clinically referred to a tertiary level service specialized in mood disorders. However, cyclothymic temperament has been previously reported, on average, in 18% of adult ADHD patients recruited in other clinical settings [25,26] and in more than half of subjects with a positive screening for ADHD in a recent epidemiological study [27].

Among cyclothymic patients recruited in our study, almost a third were diagnosed with ADHD (25 of 60, 29.4%). This figure outweighs the estimated prevalence of ADHD in adults with bipolar disorder, which nearly reaches 20% based on recent meta-analytic findings [28]. However, as far as we know, no study so far has specifically investigated the comorbidity between ADHD and cyclothymic disorder, even if symptomatologic similarities have been previously observed [20]. In addition, to the best of our knowledge, this is the first study that compared patients diagnosed with ADHD, patients diagnosed with cyclothymic disorder, and comorbid patients with both disorders.

The greatest differences were observed between non-comorbid patient groups (i.e., ADHD only vs. cyclothymia only). Cyclothymic patients were more frequently female, were older, and more frequently achieved a high educational level. ADHD patients, instead, were more frequently male, were younger, and had lower educational achievements. ADHD patients with comorbid cyclothymia showed a similarly low educational attainment, which suggests a higher impact of attentional and hyperactive–impulsive dimensions, rather than affective symptoms, on academic achievements. Indeed, even if emotional impulsivity has been previously associated with educational outcomes, including high school and college graduation, in adults with ADHD [29], attentional problems and executive dysfunctions have been repeatedly found to predict school performance and academic achievement in prospective studies conducted in children and adolescent samples [30,31,32,33].

Substance use disorders were also specifically associated with ADHD, regardless of the presence of cyclothymia. Previous studies consistently supported the association between childhood ADHD and adult substance use disorders based on both prospective assessments [34] and retrospective accounts [35]. Approximately a fourth of patients with substance use disorders are diagnosed with ADHD [36], and among patients with substance use disorders, those with ADHD are more likely to report an early onset of nicotine and illicit drug use, a reduced latency between first use and dependence/abuse, a higher severity of use, more risk behaviors, and functional impairment [37,38]. While ADHD has been implicated in the pathogenesis of substance use disorders [39], the role of cyclothymia is less clear. Cyclothymic temperamental traits have been repeatedly positively associated with substance use disorders both in clinical [40,41,42] and community settings [43,44]. However, null [45] and conflicting findings have also been reported [46] and the role of cyclothymic disorder, rather than temperament, has been understudied. Importantly, studies assessing ADHD as a potential confounder of the relationship between cyclothymia and substance use disorders are lacking. Based on our findings, we posit that the positive association between cyclothymic traits and substance use could be largely mediated by ADHD.

On the opposite, a trend toward higher comorbidity of cyclothymia with panic disorder was observed, and a first-degree family history of panic disorder, major depressive episodes, and suicide attempts were more frequently reported by cyclothymic patients. The relationship between soft bipolar disorders and panic disorder has been long established [47], and panic disorder has been proposed as a marker of genetic heterogeneity in bipolar disorder which could increase the resolution of linkage analyses [48]. In this respect, a familial subtype of bipolar disorder associated with higher rates of panic disorder, cyclothymia, and dysthymia and lower rates of substance use disorders has been isolated [48]. Panic disorder has been associated with cyclothymic and dysthymic temperamental traits in major depressive patients presenting with atypical features [49] and with short hypomanic episodes in patients with recurrent brief depressions [50]. In contrast, only a few studies have assessed panic comorbidity in adults with ADHD, and conflicting findings have emerged from comparisons with healthy controls [51,52]. In addition, panic disorder has been demonstrated as the least prevalent anxiety disorder in children with ADHD [53], and polygenic risk for ADHD has been negatively associated with panic disorder in a Japanese study [54]. Building on our results, we hypothesize that an increased rate of panic disorder could be observed in adult ADHD patients with respect to healthy controls. However, we also posit this difference to be largely explained by comorbid cyclothymia. Indeed, panic disorder in adult ADHD patients has been associated with female gender [52] and with bipolar comorbidity [55], and anxiety comorbidity in children with ADHD has been prospectively associated with bipolar disorder in a recent register-based ecological study [56].

Overall, the differences observed in familial history suggest a higher genetic load for conditions associated with negative affect and harm avoidance in patients with cyclothymia with respect to ADHD patients, whose pattern of comorbidity is indicative of a greater inclination toward sensation seeking. Indeed, ADHD has been repeatedly characterized by high novelty seeking [57], while both harm avoidance and novelty seeking coexist with cyclothymic temperament [58]. These differences are also reflected in affective temperamental traits and emotional dysregulation facet variations among patients included in our study. In comparison with ADHD patients, cyclothymic patients without ADHD were characterized by fewer hyperthymic temperamental traits, higher depressive and anxious dispositions, and greater negative emotionality and NE/PE ratio, despite similar scores on irritable temperament subscale and emotional impulsivity. These findings could be partially mediated by differences in age and gender among the groups, since women usually score lower than men in hyperthymic temperament and higher in anxious and depressive temperaments, with the latter increasing with age [59]. However, given the limited sample size, the impact of gender differences on affective temperament discrepancies could not be properly ascertained in our study.

Differences in affective temperamental traits and negative emotionality could also be attributed to different triggers for referral to psychiatric services. In our context, approximately one-fifth of patients diagnosed with ADHD during childhood or adolescence continue to use mental health services in adulthood [60], a rate similar to that observed in the United Kingdom [61]. Most patients are referred to our service for ADHD assessment due to a long-standing pattern of impulsive behavior and drug abuse. Medication resistance can also represent a major reason for referral. Finally, a subgroup of ADHD patients may access our service after repeated academic failures, occupational difficulties, and vocational impairment, which can be traced back to attentional deficits. On the other hand, cyclothymic patients mainly consult psychiatric facilities when mood and anxiety comorbidity occur [11]. Based on these assumptions, we can speculate that only a subgroup of cyclothymic subjects, predominantly characterized by anxious and dysthymic traits and problems with negative emotionality, reaches clinical attention [62], while subjects with hyperthymic variants of cyclothymia are less prone to experience significant distress due to affective fluctuations and may also show a good-to-excellent functioning [63]. These “sunny” cyclothymic conditions are unlikely to be encountered in clinical settings, unless associated with other sources of functional impairment such as ADHD. Future community studies could help to confirm or disprove this hypothesis.

Interestingly, while no significant differences among our groups were observed for cyclothymic temperament and overall negative emotional dysregulation, comorbid patients with both ADHD and cyclothymia scored the highest in these subscales. In addition, this group also showed higher affective instability with respect to ADHD patients without cyclothymia and was less frequently diagnosed with bipolar disorder type II than patients from both the other groups. This could be due to conservative attitudes toward diagnosis: since clinicians may be less prone to diagnose a patient with both ADHD and cyclothymic disorder due to overlapping symptoms of these conditions, only ADHD patients showing really severe, rapid, and frequent mood swings, which are reflected in greater affective instability scores and fail to reach duration thresholds for hypomanic episodes, may receive the diagnosis of “comorbid” cyclothymic disorder. We suggest that these patients are likely to be characterized by “dark” unstable expressions of soft bipolarity and to be diagnosed as affected by borderline personality disorder, which highly overlaps both with ADHD and bipolar disorder [64]. Indeed, it has been previously hypothesized that borderline personality traits could represent, in some cases, expressions of a developmental bipolar subtype [65,66] stemming from extreme mood lability and interpersonal sensitivity associated with cyclothymic temperament [67]. At this point, we further speculate that a considerable proportion of cases of borderline personality disorder may arise from overlapping ADHD and cyclothymia diatheses, whose expressions are mediated by developmental stages as previously hinted by Michael H. Stone [17]. While this hypothesis warrants further investigation, we are confident that a neurodevelopmentally oriented approach and a lifespan perspective could help promote accurate diagnosis and improved care of this difficult-to-treat population.

Several limitations of this study should be acknowledged. First, the clinical setting (a tertiary psychiatry unit) may have not been representative of the whole population of patients with ADHD and/or cyclothymia, but of a more complicated subpopulation, possibly showing higher rates of comorbidity and a greater severity. Second, the cross-sectional study design mostly limited the assessment of psychiatric comorbidity to retrospective accounts, which may be at risk of recall bias. In addition, the evaluation of affective temperaments and emotional dysregulation facets was based on self-report questionnaires, which may be biased by differences in social desirability, lack of insight, or malingering attempts. Finally, the criteria used to identify patients affected by cyclothymia slightly differed from DSM-5 criteria, possibly being more inclusive. As a consequence, our results should be interpreted with caution. Further longitudinal studies on epidemiological samples are needed to investigate comorbidity rates and psychopathological differences between ADHD and cyclothymia, possibly including different definitions of the latter disorder.

## 5. Conclusions

To the best of our knowledge, this is the first study to investigate the comorbidity between ADHD and cyclothymic disorder in adult clinical samples and compare patients diagnosed with either one or both of the disorders. Based on our findings, ADHD and cyclothymic disorder often co-occur. While differences can be observed in socio-demographic variables (i.e., age, gender, educational level), emotional dysregulation is a common feature in both conditions, with a slightly greater severity in patients diagnosed with both disorders. Based on psychiatric comorbidity, family history, and differences in affective temperaments and emotional dysregulation facets, cyclothymic patients were found to be significantly more prone to negative emotions, such as anxiety and depressed mood, in comparison with patients affected by ADHD, more often comorbid with substance use disorders. However, these differences could be limited to the clinical setting, where different reasons for referral are likely to bias sample selection. Patients with ADHD without emotional dysregulation and subjects with “sunny” cyclothymic temperament might be found in the community. On this assumption, we propose that the co-occurrence of conditions within the attention-deficit/hyperactivity and cyclothymic spectra can be summarized according to non-orthogonal, but also non-overlapping, intersected dimensions of deficient executive functioning and negative emotionality in adults with emotional dysregulation, both of which are likely to influence clinical attention. Alternatively, different emotional dispositions characterize patients with ADHD or cyclothymia. While positive affect could counterbalance negative emotionality in the former group, depressive and anxious tendencies may prevail in the latter.

## Figures and Tables

**Table 1:** Differences in sociodemographic variables, psychiatric comorbidity, and family history of psychiatric disorders among patients with ADHD, cyclothymia (CYC), or both (ADHD+CYC). One-way ANOVA was used for age; chi-square tests were used for other variables. F-statistics and χ^2^-statistics are reported. *p*-values < 0.05 are shown in bold.

	ADHD (N = 80)	ADHD+CYC (N = 25)	CYC (N = 60)			
**Sociodemographic variables**	**Mean (SD)/N (%)**	**Mean (SD)/N (%)**	**Mean (SD)/N (%)**	**χ^2^/F**	***p***	**Post hoc**
Age (years)	27.88 (9.69)	30.25 (12.53)	32.73 (11.74)	3.41	**0.036**	CYC > ADHD
Gender (female)	17 (21.3%)	15 (60.0%)	54 (90.0%)	65.67	**0.000**	CYC > ADHD+CYC > ADHD
Marital status				3.65	0.456	
- Unmarried	68 (85.0%)	19 (76.0%)	40 (75.5%)			
- Married/cohabiting	10 (12.5%)	4 (16.0%)	8 (15.1%)			
- Separated/divorced	2 (2.5%)	2 (8.0%)	5 (9.4%)			
Educational level				26.33	**0.000**	
- Primary/middle school	28 (35.0%)	13 (52.0%)	8 (15.1%)			ADHD, ADHD+CYC > CYC
- High school	48 (60%)	11 (44.0%)	29 (54.7%)			
- University	4 (5.0%)	1 (4.0%)	16 (30.2%)			CYC > ADHD, ADHD+CYC
Employment				4.42	0.352	
- Student	26 (32.5%)	6 (25.0%)	20 (37.7%)			
- Unemployed/housekeeper	23 (28.8%)	11 (45.8%)	12 (22.6%)			
- Employed	31 (38.8%)	7 (29.2%)	21 (39.6%)			
**Psychiatric comorbidity**						
Bipolar disorder type I	11 (13.8%)	5 (20.0%)	6 (10.0%)	1.55	0.461	
Bipolar disorder type II	37 (46.3%)	3 (12.0%)	21 (35.0%)	9.75	**0.008**	ADHD, CYC > ADHD+CYC
Substance use disorders	53 (66.3%)	14 (56.0%)	15 (28.3%)	18.59	**0.000**	ADHD, ADHD+CYC > CYC
Panic disorder	12 (15.0%)	8 (32.0%)	16 (30.2%)	5.61	0.060	
Eating disorders	17 (21.3%)	10 (40.0%)	17 (32.1%)	4.04	0.133	
**First-degree family history**						
Bipolar disorder	39 (48.8%)	11 (44.0%)	25 (48.1%)	0.18	0.916	
Major depressive disorder	4 (5.0%)	3 (12.0%)	12 (23.5%)	10.00	**0.007**	CYC > ADHD
Substance use disorders	18 (22.5%)	3 (12.0%)	4 (7.7%)	5.50	0.064	
Panic disorder	8 (10.0%)	5 (20.0%)	15 (28.8%)	7.73	**0.021**	CYC > ADHD
Eating disorders	2 (2.5%)	1 (4.0%)	3 (5.8%)	0.92	0.632	
Suicide attempts	2 (2.5%)	2 (8.0%)	8 (15.4%)	7.30	**0.026**	CYC > ADHD

**Table 2:** Differences in affective temperamental traits (TEMPS-M subscales) and emotional dysregulation facets (RIPoSt-40) among patients with ADHD, cyclothymia (CYC), or both (ADHD+CYC). One-way ANOVA results are displayed. F-statistics are reported. *p*-values < 0.05 are shown in bold.

	ADHD (N = 80)	ADHD+CYC (N = 25)	CYC (N = 60)			
**Affective temperament (TEMPS-M)**	**Mean (SD)**	**Mean (SD)**	**Mean (SD)**	**F**	***p***	**Post hoc**
Depressive	21.22 (5.72)	22.76 (5.44)	25.36 (5.96)	8.816	**0.000**	CYC > ADHD
Cyclothymic	22.63 (6.79)	26.24 (6.69)	22.92 (6.85)	2.817	0.063	
Hyperthymic	21.09 (5.62)	20.76 (7.16)	17.70 (5.94)	5.846	**0.004**	ADHD > CYC
Anxious	16.04 (5.59)	19.37 (7.19)	20.64 (6.85)	9.561	**0.000**	CYC > ADHD
Irritable	20.12 (6.88)	21.46 (7.05)	19.27 (6.35)	0.963	0.384	
**Emotional dysregulation (RIPoSt-40)**						
Affective instability	41.82 (11.75)	49.44 (12.43)	44.66 (12.81)	3.837	**0.024**	ADHD+CYC > ADHD
Positive emotionality	40.38 (8.52)	42.48 (10.21)	39.85 (9.25)	0.758	0.470	
Negative emotionality	39.15 (9.75)	39.56 (10.77)	44.56 (9.23)	5.723	**0.004**	CYC > ADHD
Emotional impulsivity	30.79 (8.60)	31.16 (9.00)	29.40 (7.82)	0.453	0.636	
Negative emotional dysregulation	111.35 (25.80)	120.16 (29.43)	118.62 (24.87)	1.844	0.161	
Negative/positive emotionality ratio	1.00 (0.35)	1.03 (0.63)	1.22 (0.55)	3.532	**0.032**	CYC > ADHD

**Table 3:** **Principal component analysis results (two-component solution).** Component loadings of each item in each component are shown. Component loadings > 0.4 are shown in bold.

	C1. Negative Affect Dysregulation	C2. Positive Affect
**Affective temperament (TEMPS-M)**		
Depressive	**0.78**	−0.21
Cyclothymic	**0.76**	0.32
Hyperthymic	−0.19	**0.87**
Anxious	**0.68**	0.04
Irritable	**0.62**	**0.44**
**Emotional dysregulation (RIPoSt-40)**		
Affective instability	**0.83**	0.15
Positive emotionality	0.21	**0.67**
Negative emotionality	**0.85**	−0.23
Emotional impulsivity	**0.73**	0.24
Eigenvalue	4.07	1.67
Variance (%)	45.18	18.56

Abbreviations: C = component.

## Data Availability

Data are available on request from the corresponding author.
